# Maintenance BEZ235 Treatment Prolongs the Therapeutic Effect of the Combination of BEZ235 and Radiotherapy for Colorectal Cancer

**DOI:** 10.3390/cancers11081204

**Published:** 2019-08-19

**Authors:** Yu-Hsuan Chen, Chun-Wei Wang, Ming-Feng Wei, Yi-Shin Tzeng, Keng-Hsueh Lan, Ann-Lii Cheng, Sung-Hsin Kuo

**Affiliations:** 1 Department of Oncology, National Taiwan University Hospital, Taipei 10002, Taiwan; 2 Cancer Research Center, College of Medicine, National Taiwan University, Taipei 10617, Taiwan; 3 Graduate Institute of Oncology, College of Medicine, National Taiwan University, Taipei 10617, Taiwan; 4 National Taiwan University Cancer Center, College of Medicine, National Taiwan University, Taipei 10617, Taiwan; 5 Department of Internal Medicine, National Taiwan University Hospital, Taipei 10002, Taiwan

**Keywords:** PI3K/mTOR, BEZ235, radiation, colorectal cancer, maintenance

## Abstract

Our previous study demonstrated that administration of NVP-BEZ235 (BEZ235), a dual PI3K/mTOR inhibitor, before radiotherapy (RT) enhanced the radiotherapeutic effect in colorectal cancer (CRC) cells both in vitro and in vivo. Here, we evaluated whether maintenance BEZ235 treatment, after combinatorial BEZ235 + RT therapy, prolonged the antitumor effect in CRC. K-RAS mutant CRC cells (HCT116 and SW480), wild-type CRC cells (HT29), and HCT116 xenograft tumors were separated into the following six study groups: (1) untreated (control); (2) RT alone; (3) BEZ235 alone; (4) RT + BEZ235; (5) maintenance BEZ235 following RT + BEZ235 (RT + BEZ235 + mBEZ235); and (6) maintenance BEZ235 following BEZ235 (BEZ235 + mBEZ235). RT + BEZ235 + mBEZ235 treatment significantly inhibited cell viability and increased apoptosis in three CRC cell lines compared to the other five treatments in vitro. In the HCT116 xenograft tumor model, RT + BEZ235 + mBEZ235 treatment significantly reduced the tumor size when compared to the other five treatments. Furthermore, the expression of mTOR signaling molecules (p-rpS6 and p-eIF4E), DNA double-strand break (DSB) repair-related molecules (p-ATM and p-DNA-PKcs), and angiogenesis-related molecules (VEGF-A and HIF-1α) was significantly downregulated after RT + BEZ235 + mBEZ235 treatment both in vitro and in vivo when compared to the RT + BEZ235, RT, BEZ235, BEZ235 + mBEZ235, and control treatments. Cleaved caspase-3, cleaved poly (ADP-ribose) polymerase (PARP), 53BP1, and γ-H2AX expression in the HCT116 xenograft tissue and three CRC cell lines were significantly upregulated after RT + BEZ235 + mBEZ235 treatment. Maintenance BEZ235 treatment in CRC cells prolonged the inhibition of cell viability, enhancement of apoptosis, attenuation of mTOR signaling, impairment of the DNA-DSB repair mechanism, and downregulation of angiogenesis that occurred due to concurrent BEZ235 and RT treatment.

## 1. Introduction

Preoperative radiotherapy (RT) is used to reduce the risk of local recurrence and improve survival in patients with locally advanced rectal cancer [1,2,3]. Adding chemotherapy to RT in the neoadjuvant setting for locally advanced rectal cancer provides superior local control and a higher anal preservation rate as well as less treatment-related toxicity when compared with postoperative concurrent chemoradiotherapy (CCRT) [4,5]. In these combined modalities, conventionally fractionated long-course RT (1.8 Gy × 25–28 fractions) is commonly prescribed, and the operation is performed 6–8 weeks later, after the down-staging of the tumor and recovery of the irradiated tissue from the acute radiation reaction [4,5]. The mechanism of cell death from radiation is mitotic death; therefore, rectal tumors need time to achieve better tumor regression rates after neoadjuvant CCRT. However, a proportion of patients with locally advanced rectal cancer may be at risk of metastatic progression during the 6–8-week period before the operation when no treatment is given.

Randomized trials have reported that, in addition to conventional long-course RT, hypofractionated short-course RT (5 Gy × 5 fractions) followed immediately by the operation is another option for locally advanced rectal cancer [6,7]. In two ongoing randomized trials comparing different treatment regimens for neoadjuvant short-course RT in patients with rectal cancer, more satisfactory pathological complete remission (pCR) rates and clinical outcomes have been observed in patients undergoing systemic chemotherapy following short-course RT than in those not undergoing systemic chemotherapy [8,9]. These results suggest that when microscopic dissemination is limited by systemic chemotherapy, delaying surgery after short-course RT provides time for tumor regression and increases the pCR rate [10,11,12].

Mutation of K-RAS occurs in 30–50% of CRC cells and K-RAS mutations can lead to the aberrant activation of several oncogenic signaling pathways including the phosphoinositide 3-kinase (PI3K)/AKT/mammalian target of the rapamycin (mTOR) signaling pathway [13,14]. The PI3K/AKT/mTOR signaling pathway plays a crucial role in promoting cellular proliferation, increasing angiogenesis, attenuating apoptosis, and fostering metastasis of CRC cells [15,16,17,18]. Konstantinidou et al. showed that NVP-BEZ235 (BEZ235) increased the proapoptotic effects of RT both in oncogenic K-RAS-expressing non-small cell lung cancer cell (NSCLC) lines and in transgenic mice harboring oncogenic K-RAS-induced NSCLC [19].

In the PI3K/AKT-signaling family, ataxia telangiectasia mutated (ATM) and DNA-dependent protein kinase catalytic subunit complex (DNA-PKcs) are two major kinases that participate in the important DNA double strand break (DNA-DSB) repair pathways [20,21]. Considering that DNA-DSB is the most lethal type of cell death after radiation, the activation of the PI3K/AKT/mTOR pathway can endorse the resistance of cancer cells to RT through enhancing DNA damage repair [22,23,24].

Several studies have demonstrated that BEZ235, a dual PI3K and mTOR inhibitor, enhanced radiosensitivity in glioblastoma cells, laryngeal cancer cells, and hypopharyngeal cancer cells through the inhibition of ATM and DNA-PKcs-associated DNA repair [25,26,27]. In addition, BEZ235 has been reported to inhibit cell viability and enhance the radiosensitivity of colorectal cancer (CRC) both in vitro and in vivo through the attenuation of radiation-inducing AKT/mTOR signaling activation and the inhibition of the DNA-DSB repair mechanism [28]. The BEZ235 also significantly downregulated the radiation-induced VEGF expression in lung cancer cell lines [29].

As the histological manifestations and biological behaviors are similar between rectal cancer and colon cancer and no purely rectal cancer cell lines can be obtained from the American Type Culture Collection (ATCC), we selected three colon cancer cell lines, HCT116 (K-RAS mutation; p53 wild type), HT29 (K-RAS wild type; p53 mutation), and SW480 (K-RAS mutation; p53 mutation) from the ATCC to mimic the rectal cancer environment in vitro [30] and assess whether maintenance BEZ235 can prolong the therapeutic effects of combined RT and BEZ235 in a neoadjuvant setting of rectal cancer. This study investigated the radiotherapeutic effects and molecular mechanisms of maintenance BEZ235 therapy following BEZ235 and RT treatment in three CRC cell lines (HCT116, HT29, and SW40) and in a HCT116 xenograft model.

## 2. Results

### 2.1. Maintenance BEZ235 Treatment Following Concurrent BEZ235 and RT Treatment Inhibited Cell Viability and Increased Apoptosis in CRC Cells

**Figure 1:**
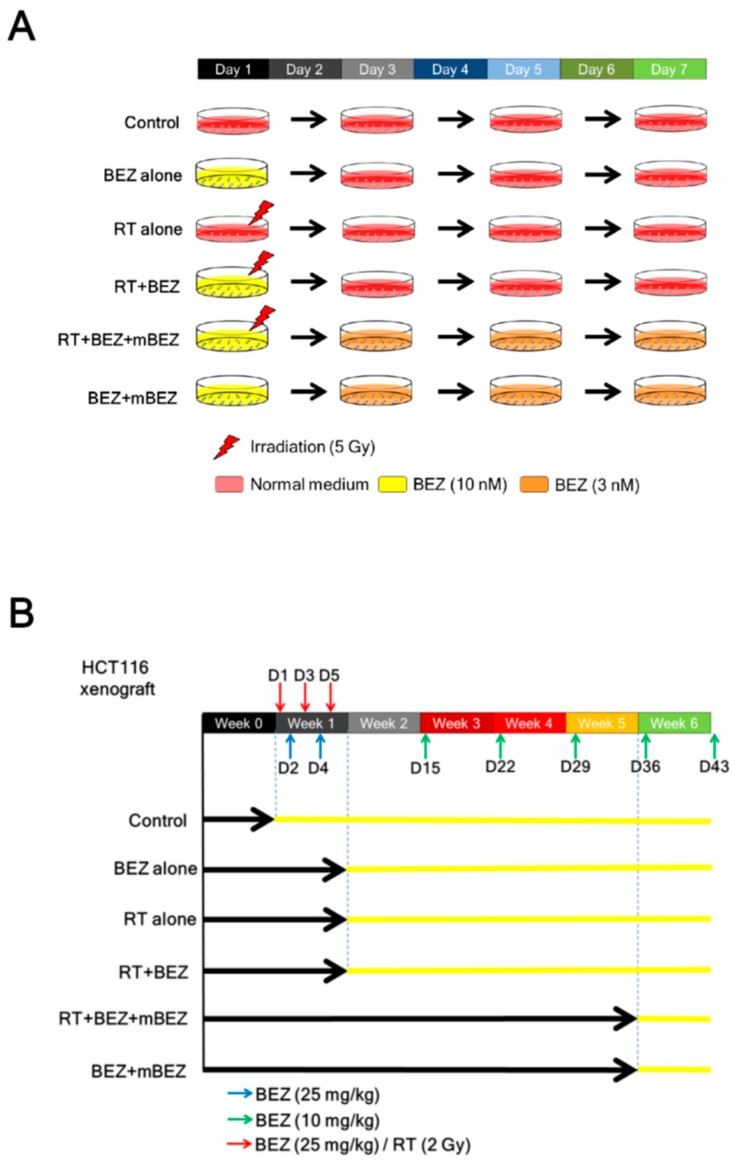
Schematic diagram of the therapeutic protocol. (**A**) In vitro experiments. CRC cells were separated into six groups: control group (untreated), NVP-BEZ235 alone group (BEZ alone), radiotherapy (RT) alone group (RT alone), RT + BEZ235 group (RT + BEZ), RT + BEZ235 + maintenance BEZ235 group (RT + BEZ + mBEZ), and BEZ235 + maintenance BEZ235 (BEZ + mBEZ). (**B**) In vivo experiments. Immunocompromised mice were subcutaneously injected with 1 × 10^6^ HCT116 cancer cells and then divided into six groups.

To evaluate the antitumor effect of maintenance BEZ235 treatment following a combination of BEZ235 + RT treatment, we designed in vitro and in vivo experimental groups according to the illustration in Figure 1.

**Figure 2:**
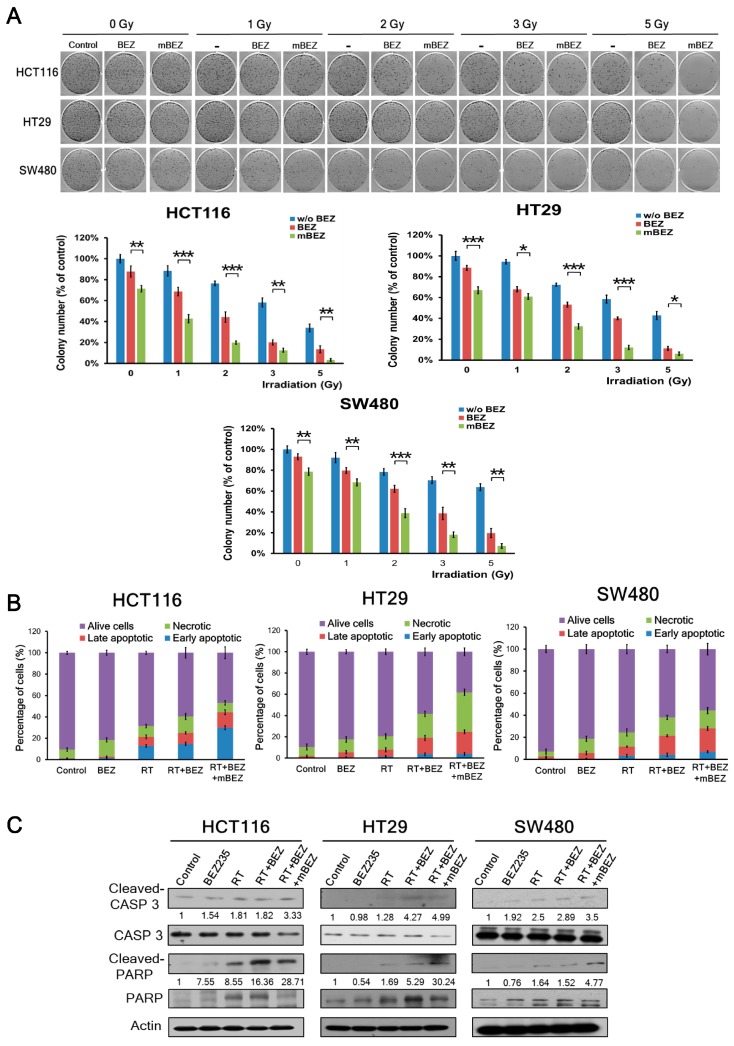
BEZ235 maintenance treatment following RT + BEZ235 treatment resulted in significantly less cell survival and more apoptosis markers in CRC cells. (**A**) In three CRC cell lines (HCT116, HT29, and SW480), a colony formation study (1000 cells per well) showed that the rate of cell survival significantly decreased after RT + BEZ235 + mBEZ235 treatment. In addition, RT + BEZ235 + mBEZ235 treatment exhibited a RT dose-dependent reduction in clonogenic survival fraction. The results are expressed as the mean ± standard error (SE) of three experiments. * *p* < 0.05; ** *p* < 0.01; *** *p* < 0.001. (**B**) Quantification of apoptosis via annexin V-propidium iodide staining of the HCT116, HT29, and SW480 cells after various treatments. Completely apoptotic cells were more prominently found after RT + BEZ235 + mBEZ235 treatment. (**C**) Western blotting showed that BEZ235 maintenance treatment substantially enhanced the level of apoptosis (cleaved caspase-3 [CASP 3] and cleaved PARP) induced by radiation. PARP, CASP 3, and β-actin served as the controls. The band intensities were analyzed by ImageJ software. The relative ratios of the cleaved protein to non-cleaved protein amounts were quantified and indicated underneath each gel. The relative ratio of the control group is arbitrarily presented as 1.

This clonogenic assay investigated whether RT + BEZ235 + mBEZ235 treatment could predominantly inhibit the cell viability of three CRC cells compared with that on RT + BEZ235, RT, BEZ235, BEZ235 + mBEZ235, and the control treatments. In the clonogenic assay with HCT116, HT29, or SW480 cells with 1000 cells seeded per well, we found that RT + BEZ235 treatment followed by BEZ235 maintenance treatment significantly inhibited cell proliferation when compared to treatment without maintenance therapy (Figure 2A). The significant inhibitory effects of RT + BEZ235 treatment followed by BEZ235 maintenance treatment were found at different RT doses (1 Gy, 2 Gy, 3 Gy, and 5 Gy) compared to the RT + BEZ235 treatment (Figure 2A). In addition, BEZ235 + mBEZ235 significantly inhibited the proliferation of three CRC cells compared to the BEZ235 alone group (at RT 0 Gy, Figure 2A). As shown in Supplementary Figure S1A,B (the clonogenic assay by seeding 200 cells or 500 cells per well), RT + BEZ235 + mBEZ235 treatment significantly inhibited cell proliferation when compared with the RT + BEZ235, RT, BEZ235, and control treatments.

Annexin V staining was used to detect CRC cell apoptosis after each treatment. BEZ235 treatment alone did not induce much apoptosis in three CRC cells when compared to the control (Figure 2B). In the RT alone group, annexin V staining revealed 21.31%, 7.89%, and 11.57% apoptosis in the HCT116, HT29, and SW480 cells, respectively. After RT + BEZ235 treatment, annexin V staining revealed 25.11%, 18.93%, and 21.37% apoptosis in the HCT116, HT29, and SW480 cells, respectively (Figure 2B and Supplementary Figure S2A). In the RT + BEZ235 + mBEZ235 treatment group, annexin V staining revealed 44.34%, 24.63%, and 28.03% apoptosis in the HCT116, HT29, and SW480 cells, respectively (Figure 2B and Supplementary Figure S2A). RT + BEZ235 treatment increased the total number of apoptotic events to 3.80%, 11.04%, and 9.80%, in the HCT116, HT29, and SW480 cells, respectively, when compared to RT alone. Furthermore, RT + BEZ235 + mBEZ235 treatment increased apoptosis to 19.23%, 5.70%, and 6.66% in the HCT116, HT29, and SW480 cells, respectively, when compared to RT + BEZ235. In the BEZ235 + mBEZ235 treatment, annexin V staining revealed 12.89%, 13.35%, and 6.35% apoptosis in the HCT116, HT29, and SW480 cells, respectively (Supplementary Figure S2B). BEZ235 + mBEZ235 treatment still increased the total number of apoptosis events in three CRC cells when compared to BEZ235 (Supplementary Figure S2A,B). These findings suggest that BEZ235 can increase RT-induced apoptosis, and continuous treatment with BEZ235 still increased apoptosis in these three CRC cell lines.

We found that the level of cleaved caspase-3 and poly (ADP-ribose) polymerase (PARP) was significantly upregulated in HCT116 cells treated with RT + BEZ235 + mBEZ235 when compared to those treated with BEZ235, RT, RT + BEZ235, and BEZ235 + mBEZ235 (Figure 2C and Supplementary Figure S3A). Similar results were observed in the HT29 cells and SW480 cells (Figure 2C and Supplementary Figure S3A). Taken together, these findings suggest that RT + BEZ235 + mBEZ235 treatment caused increased apoptosis when compared to RT + BEZ235 treatment in all CRC cell lines.

### 2.2. Maintenance BEZ235 Treatment Following RT + BEZ235 Treatment Enhanced CRC Cell Treatment Effects through Attenuating mTOR Signaling and Inhibiting Angiogenesis-Related Molecules

**Figure 3:**
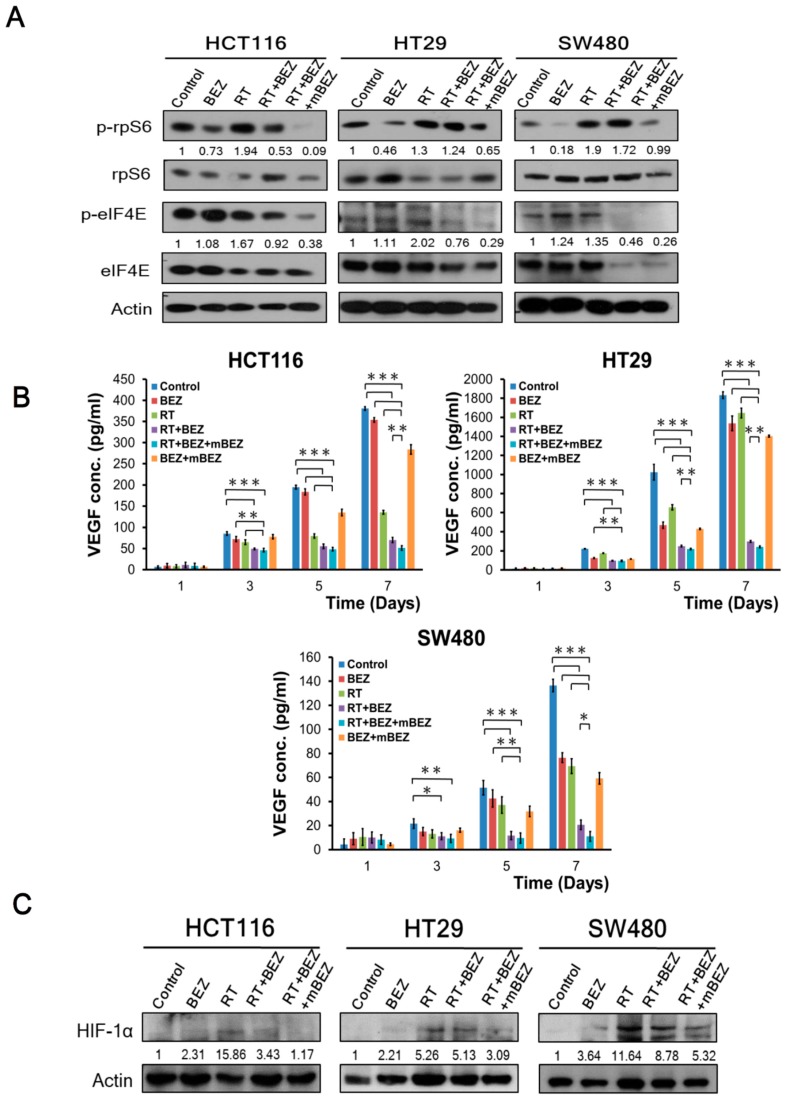
BEZ235 maintenance treatment following RT + BEZ235 treatment sensitized CRC cells to radiation by attenuating mTOR signaling- and angiogenesis-associated molecules. (**A**) BEZ235 maintenance treatment caused a marked decrease in radiation-induced phosphorylation levels of rpS6 and eIF4E in the CRC cells. The relative amounts of phosphorylated and non-phosphorylated proteins were quantified. The relative ratio of the control group was defined as 1. (**B**) VEGF-A concentrations were analyzed via enzyme-linked immunosorbent assay (ELISA). The results are expressed as the mean ± standard error (SE) of three experiments. * *p* < 0.05; ** *p* < 0.01; *** *p* < 0.001. (**C**) BEZ235 maintenance treatment significantly inhibited HIF-1α expression when compared with RT and RT + BEZ235 treatment in three CRC cells. The relative amounts of phosphorylated and non-phosphorylated proteins were quantified. The relative ratio of the control group was defined as 1.

As shown in Figure 3A, we found that RT alone upregulated p-rpS6 and p-e-IF4E in the HCT116 cells, HT29 cells, and SW480 cells. In comparison, we found that the RT + BEZ235 and RT + BEZ235 + mBEZ235 regimens decreased phosphorylation of rpS6 and e-IF4E in all three cell lines. In addition, BEZ235 + mBEZ235 treatment suppressed the phosphorylation of rpS6 and e-IF4E in all three cell lines when compared with BEZ235 alone (Figure 3A and Supplementary Figure S3B).

Furthermore, when compared with the RT + BEZ235 treatment group, RT + BEZ235 + mBEZ235 treatment significantly reduced the phosphorylation of rpS6 by 83% in HCT116 cells, 48% in HT29 cells, and 50% in SW480 cells (Figure 3A). Similarly, RT + BEZ235 + mBEZ235 treatment significantly reduced the phosphorylation of e-IF4E by 59% in HCT116 cells, 62% in HT29 cells, and 44% in SW480 cells when compared with phosphorylation levels after RT + BEZ235 treatment (Figure 3A).

Angiogenesis is crucial for tumor growth and metastasis in CRC [31,32]. To explore whether the BEZ235 maintenance treatment can significantly inhibit angiogenesis, we assessed VEGF-A expression via ELISA in three CRC cell lines. As shown in Figure 3B, on Day 7, the RT + BEZ235 treatment group had significantly reduced VEGF-A levels when compared to the RT alone, BEZ235 alone, BEZ235 + mBEZ235, and control groups in three CRC cell lines. Importantly, the RT + BEZ235 + mBEZ235 treatment group significantly reduced VEGF-A expression when compared with the RT + BEZ235 treatment group in HCT116 cells. Similar findings were also found in the HT29 and SW480 cells (Figure 3B).

Hypoxia-inducible factor-1α (HIF-1α) plays a critical role in response to tumor hypoxia and mediates tumor resistance to chemotherapy and radiotherapy [33,34]. HIF-1α is a key angiogenesis regulatory factor for CRC cells [35]. In addition to VEGF-A, immunoblotting revealed that HIF-1α expression was significantly downregulated after RT + BEZ235 + mBEZ235 treatment when compared with the RT and RT + BEZ235 treatments in all CRC cells (HCT116, HT29, and SW480 cells) (Figure 3C). Interestingly, BEZ235 + mBEZ235 treatment significantly downregulated the expression of HIF-1α when compared with BEZ235 alone group in all CRC cells (HCT116, HT29, and SW480 cells) (Figure 3C and Supplementary Figure S3C).

### 2.3. BEZ235 Maintenance Treatment Following RT + BEZ235 Treatment Enhanced Antitumor Effects through Inhibiting the DNA-DSB Repair Pathway, and Increasing DNA Damage in CRC Cells

**Figure 4:**
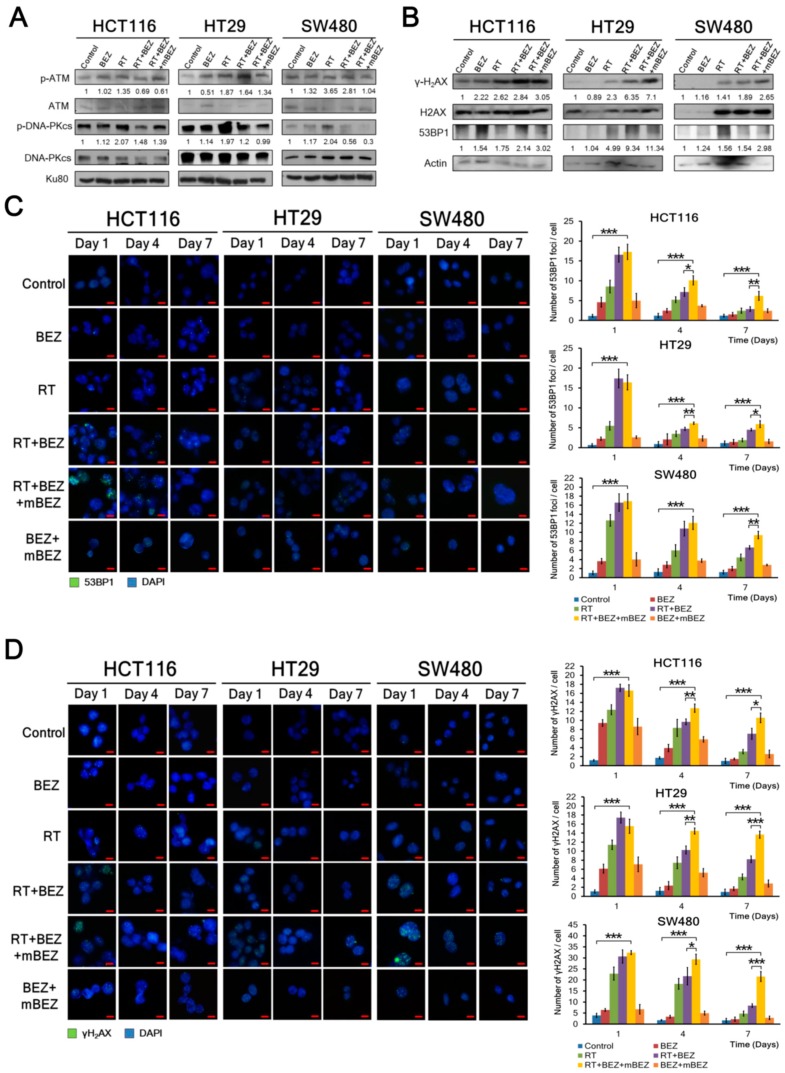
BEZ235 maintenance treatment following RT + BEZ235 treatment increased the susceptibility of CRC cells to radiation by inhibiting DNA repair mechanisms and increasing the effects of DNA damage. (**A**) BEZ235 maintenance treatment following RT + BEZ235 treatment significantly inhibited p-ATM and p-DNA-PKcs in three CRC cell lines compared to the BEZ235, RT, RT + BEZ235, and control treatments. The relative amounts of phosphorylated and non-phosphorylated proteins were quantified. The relative ratio of the control group was defined as 1. (**B**) RT + BEZ235 treatment followed by BEZ235 maintenance treatment significantly increased γ-H2AX and 53BP1 expression in three CRC cell lines compared to the BEZ235, RT, RT + BEZ235, and control treatments. (**C**) Cells were treated as described in Figure 1A and labeled with anti-53BP1 primary antibody and DyLight 488-conjugated secondary antibody on days 1, 4, and 7 of the treatment period. The 53BP1 foci were observed using immunofluorescence with a fluorescence microscope. Nuclei were counterstained with DAPI. Immunofluorescence analysis (left panel) accompanied by quantification analysis (right panel) showed that BEZ235 maintenance treatment showed prolonged DNA damage (more 53BP1 foci) in three CRC cell lines until day 7 when compared to the other treatment groups. The number of 53BP1 foci was counted using 20 cells for each treatment. * *p* < 0.05; ** *p* < 0.01; *** *p* < 0.001. Scale bar: 10 μm. (**D**) Cells were treated as described in Figure 1A and labeled with the anti-γ-H2AX primary antibody and DyLight 488-conjugated secondary antibody. The amount of γ-H2AX was maintained at a consistent level after BEZ235 maintenance treatment until day 7 when compared to the RT + BEZ235, RT, BEZ235, BEZ235 + mBEZ235, and control treatments in all three CRC cells. The amount of γ-H2AX was counted using 20 cells for each condition. * *p* < 0.05; ** *p* < 0.01; *** *p* < 0.001. Scale bar: 10 μm.

Furthermore, we found that RT treatment upregulated DNA repair mechanisms through the p-ATM and p-DNA-PKcs markers in all three CRC cell lines, whereas BEZ235 treatment decreased the RT-induced expression of p-ATM and p-DNA-PKcs in all CRC cell lines (Figure 4A). When compared with RT + BEZ235 treatment alone, post-treatment BEZ235 maintenance treatment significantly downregulated p-ATM and p-DNA-PKcs expression by 12% and 7% in the HCT116 cells (K-RAS mutation; p53 wild type), 18% and 18% in the HT29 cells (K-RAS wild type; p53 mutation), and 63% and 47% in the SW480 cells (K-RAS mutation; p53 mutation), respectively (Figure 4A). Like the BEZ235 alone group, BEZ235 + mBEZ235 treatment did not significantly alter the expression of p-ATM and p-DNA-PKcs (Supplementary Figure S3D).

In addition to p-ATM and p-DNA-PKcs, we assessed whether BEZ235 maintenance treatment could increase the expression of 53BP1, which plays an important role in DNA damage responses [35], and γ-H2AX, a marker of DNA double strand breaks [36], when compared with the other five treatment groups. We found that BEZ235 and RT treatments upregulated 53BP1 and γ-H2AX more than RT, BEZ235, BEZ235 + mBEZ235, and the control (Figure 4B and Supplementary Figure S3E). When compared with RT + BEZ235 treatment alone, post-treatment BEZ235 maintenance treatment significantly increased 53BP1 and γ-H2AX expression by 41% and 7% in the HCT116 cells, 21% and 12% in the HT29 cells, and 93% and 40% in the SW480 cells, respectively (Figure 4B).

To observe whether 53BP1 and γ-H2AX expression can be maintained during BEZ235 maintenance treatment, we used an immunofluorescence assay to assess the amount of 53BP1 and γ-H2AX foci per cell on days 1, 4, and 7. Cells were treated according to the description in Figure 1A. As shown in Figure 4C, we found that RT + BEZ235 treatment followed by BEZ235 maintenance treatment showed prolonged DNA damage (more 53BP1 foci) in three CRC cell lines until day 7 when compared with the RT + BEZ235, RT, BEZ235, BEZ235 + mBEZ235, and control treatments. Similarly, the amount of γ-H2AX was significantly prolonged by BEZ235 maintenance treatment until day 7 when compared with that by the other five treatment groups in all CRC cell lines (Figure 4D).

### 2.4. BEZ235 Maintenance Treatment Following RT + BEZ235 Treatment Increased CRC Tumor Suppressive Effect In Vivo

**Figure 5:**
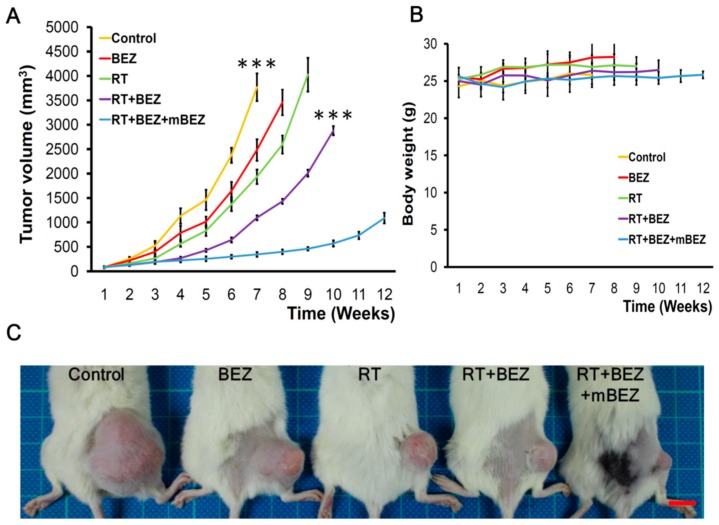
BEZ235 maintenance treatment following RT + BEZ235 treatment significantly inhibited CRC xenograft tumor growth. (**A**) Maintenance BEZ235 following RT + BEZ235 treatment significantly suppressed xenograft tumor growth when compared with other treatment groups, especially RT + BEZ235 treatment without BEZ235 maintenance. The results of the tumor volume are expressed for *n* = 6 in each treatment group. *** *p* < 0.001 compared with the control group; *** *p* < 0.001 compared with the RT + BEZ235 group. (**B**) The body weight of mice in the five groups did not differ, and no marked changes were observed during the treatment period. (**C**) Representative pictures of each treatment group. Mice were photographed during the seventh week after treatment. Scale bar: 10 mm.

Next, we evaluated the antitumor effect of concurrent RT + BEZ235 with or without maintenance BEZ235 treatment in a HCT116 CRC xenograft tumor model. We found that on day 43 after the initial treatment, the tumor growth in the RT + BEZ235 + mBEZ235 treatment group was significantly inhibited when compared with that in the untreated control group (*p* < 0.001), BEZ235 alone group, BEZ235 + mBEZ235 group, and RT alone group (Figure 5A,C and Supplementary Figure S4A,C). In addition, on day 43, the RT + BEZ235 + mBEZ235 treatment group mice had tumors 69% smaller than those in the RT + BEZ235 treatment group (*p* < 0.001, Figure 5A,C). As shown in Figure 5A, we found that the antitumor effect of RT + BEZ235 + mBEZ235 (10 mg/kg/d for five days) lasted for 10 weeks and up to 11 weeks after starting treatment, indicating that mBEZ235 can prolong antitumor control, which resulted from RT combined with BEZ235. As shown in Figure 1B (treatment schematic diagram), the treatment was tolerated in mice during and after concurrent RT + BEZ235 and maintenance BEZ235 treatments (Figure 5B and Supplementary Figure S4B).

### 2.5. BEZ235 Maintenance Treatment Following RT + BEZ235 Treatment Upregulated Apoptosis- and DNA Damage-Related Molecules and Downregulated the Expression of mTOR Signaling-, DNA-DSB-, and Angiogenesis-Related Molecules In Vivo

**Figure 6:**
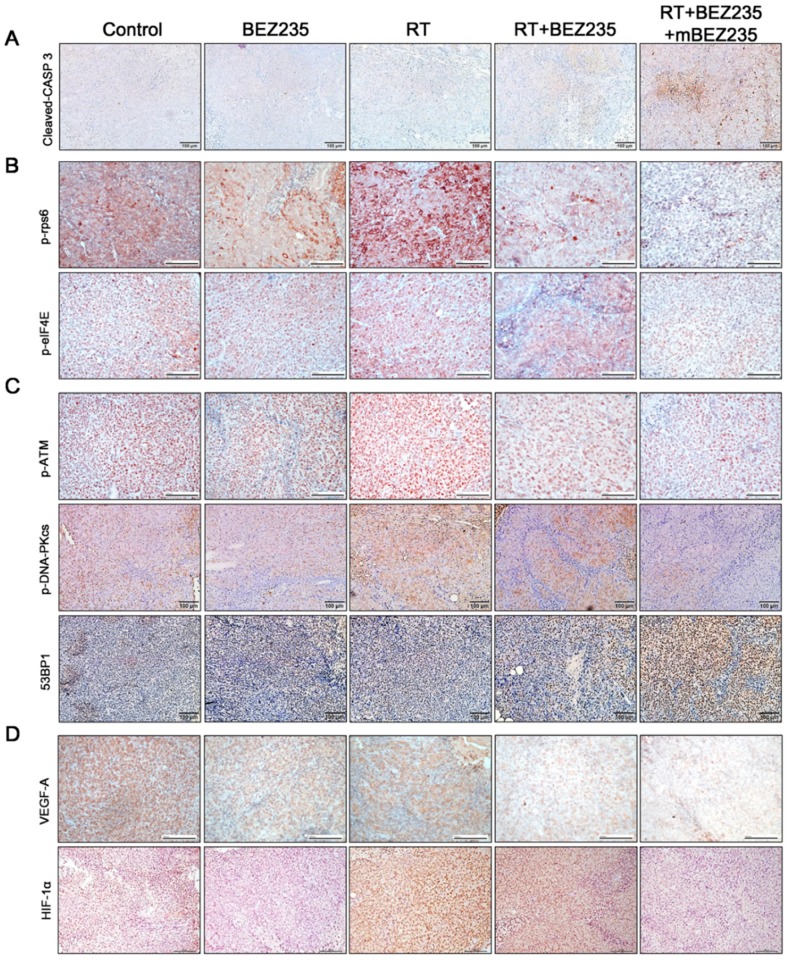
Expression of the apoptosis-, mTOR signaling pathway-, DNA-DSB repair-, DNA damage-, and angiogenesis-related molecules in CRC xenograft tissue following each treatment by immunohistochemistry. (**A**) BEZ235 maintenance treatment following RT + BEZ235 treatment (RT + BEZ235 + mBEZ235) significantly increased cleaved caspase-3 (CASP 3) expression when compared with other four treatments on day 43. (**B**) p-rpS6 and p-eIF4E expression after RT + BEZ235 + mBEZ235 treatment was downregulated when compared with those after RT + BEZ235 or control treatment on day 43. (**C**) RT + BEZ235 + mBEZ235 treatment significantly downregulated p-ATM and p-DNA-PKcs expression when compared with RT + BEZ235 and control treatment on day 43, whereas 53BP1 expression was significantly increased after RT + BEZ235 + mBEZ235 treatment. (**D**) RT + BEZ235 + mBEZ235 treatment significantly downregulated VEGF-A and HIF-1α when compared with other treatments on day 43. Scale bar = 100 μm.

Immunohistochemical staining of HCT116 xenograft tissue showed that RT + BEZ235 + mBEZ235 treatment upregulated the expression of cleaved caspase-3 when compared with the RT + BEZ235, RT, BEZ235, BEZ235 + mBEZ235, and control treatments (Figure 6A and Supplementary Figure S5A). As shown in Figure 6B,C increased p-rps6, p-eIF4E, p-ATM, and p-DNA-PKcs expression was found after RT treatment when compared with the control. In contrast with the RT treatment, the BEZ235 alone, BEZ235 + mBEZ235 and RT + BEZ235 treatments resulted in decreased p-rpS6, p-eIF4E, p-ATM, and p-DNA-PKcs expression (Figure 6B,C and Supplementary Figure S5A). Furthermore, p-rpS6, p-e-IF4E, p-ATM, and p-DNA-PKcs expression was significantly attenuated in tumor cells after RT + BEZ235 + mBEZ235 treatment when compared with those after RT + BEZ235 treatment. RT + BEZ235 + mBEZ235 treatment also significantly enhanced 53BP1 expression when compared with the RT + BEZ235, RT, BEZ235, BEZ235 + mBEZ235, and control treatments (Figure 6C and Supplementary Figure S5A).

Immunohistochemistry analysis showed that VEGF-A and HIF-1α expression was downregulated following RT + BEZ235 + mBEZ235 treatment when compared with the RT + BEZ235, RT, BEZ235, BEZ235 + mBEZ235, and control treatments (Figure 6D and Supplementary Figure S5A). Protein expression levels of cleaved caspase-3, p-rps6, p-eIF4E, p-ATM, p-DNA-PKcs, 53BP1, VEGF-A, and HIF-1α in CRC xenograft tumor cells following each treatment were quantified as shown in Supplementary Figure S5B. Both the protein expression levels and immunohistochemical analysis (Figure 6 and Supplementary Figure S5A) showed similar findings.

In addition to the immunohistochemical analysis, we used Western blotting to assess the protein level changes in the HCT116 xenograft tissue after each treatment. We showed that the levels of proteins involved in mTOR signaling (p-rps6 and p-eIF4E), DNA repair (p-ATM and p-DNA-PKcs), and angiogenesis (VEGF-A and HIF-1α) were significantly downregulated in tumors treated with RT + BEZ235 + mBEZ235 when compared with those treated with RT + BEZ235 (Supplementary Figure S6). In contrast, the levels of cleaved caspase-3, 53BP1, and γ-H2AX were upregulated in tumor cells treated with RT + BEZ235 + mBEZ235 when compared with those treated with RT + BEZ235 (Supplementary Figure S6).

## 3. Discussion

In this study, we observed that maintenance BEZ235 treatment following concurrent BEZ235 + RT treatment prolonged the therapeutic effect in three CRC cell lines with different K-RAS or p53 mutations in an in vitro study. The enhanced therapeutic effects of maintenance BEZ235 treatment following concurrent BEZ235 + RT treatment was further observed in HCT116 xenograft tumors in vivo.

The prolonged antitumor effects of BEZ235 maintenance treatment in CRC cell lines and HCT116 xenograft tumor tissue are mainly attributed to the continuous downregulation of molecules related to mTOR signaling (rpS6 and eIF4E) and the DNA-DSB repair pathway (ATM and DNA-PKcs) resulting from concurrent BEZ235 and RT treatment. BEZ235 maintenance treatment increased 53BP1 and γ-H2AX production, indicating that persistent DNA damage is another mechanism contributing to the enhanced antitumor effects. Our findings agree with the results from Gil del Alcazar and coworkers that BEZ235 treatment can attenuate ionizing radiation-induced DNA repair through the downregulation of both DNA-PKcs and ATM kinases and the upregulation of 53BP1, thus prolonging the survival of glioblastoma tumor-bearing mice [25]. Mukherjee and coworkers also reported that BEZ235 treatment increased the radiosensitivity of glioblastoma both in vitro and in vivo through inhibition of the DNA repair pathway and escalating DNA damage [26]. In head and neck and bladder cancer cell lines, Fokas and coworkers reported that BEZ235 treatment prolonged ionizing radiation-induced DNA damage, as evidenced by the increased production of γ-H2AX, and enhanced the antivascular effect of RT by attenuating VEGF-associated effects [27].

Mutations in p53 can deregulate the p53-signaling pathway and contribute to the tumorigenesis of CRC cells [37]. Upstream from RT-induced DNA damage, wild-type p53 can suppress mTOR activity in CRC cells [38,39,40]. In the current study, we found that BEZ235 treatment increased the radiosensitivity of three CRC cell lines with either wild type p53 (HCT116) or mutant p53 (HT29 and SW480). Miyasaka and coworkers also found that BEZ235 treatment increased the radiosensitivity of endometrial cancer cells irrespective of wild-type and mutant p53 status [41]. In p53 mutant CRC cells, Massey and coworkers reported the BEZ235-induced synergistic cytotoxicity of the Chk1 inhibitor V158411 and concluded that the mechanism occurred via DNA-PKcs downregulation involving homologous recombination repair [42]. These findings suggest that BEZ235 treatment not only inhibits AKT/mTOR signaling, but also attenuates DNA-DSB repair, thus contributing to increased radiosensitivity in wild and mutant p53 CRC cell lines.

In addition to the DNA-DSB repair pathway, angiogenesis is biologically associated with radioresistance in a variety of solid tumors [43]. VEGF-A, the most critical angiogenic growth factor, is involved in the angiogenesis of various cancers including CRC [44]. Two randomized trials have reported that, compared with chemotherapy alone, adding bevacizumab (anti-VEGF-A) to chemotherapy prolonged the progression-free survival rate and overall survival in patients with metastatic CRC [45,46]. In addition to its pivotal role in hypoxia-mediated radioresistance, HIF-1α promotes tumor recurrence after RT by increasing tumor repopulation and protecting tumor blood vascular structure via VEGF production [34,47]. Indeed, the mTOR signaling pathway not only increases HIF-1α synthesis, but also promotes tumor angiogenesis in CRC cells [48,49,50]. In this study, we observed that BEZ235 and RT treatments followed by BEZ235 maintenance treatment significantly downregulated VEGF-A and HIF-1α expression in CRC cell lines and xenograft tumor tissue. These results revealed that the extended inhibition of angiogenesis and HIF-1α were other crucial mechanisms contributing to the increased radiosensitivity and prolonged antitumor effects of BEZ235 maintenance treatment.

Previous studies suggested that the activated PI3K/AKT/mTOR signaling pathway can promote cell survival and cancer cell growth by attenuating apoptosis-related signaling, and this effect can result in radiotherapy resistance [51]. Chen et al. found that the PI3K inhibitor LY294002 decreased tumor tumorigenicity in CRC stem cells by increasing production of the apoptosis-related protein cleaved caspase-3 [52]. Another study also showed that BEZ235 alone can decrease colon cancer cell viability via apoptosis, and a combination of nanoparticles of layered double hydroxide loaded with fluorouracil with BEZ235 produced a greater effect on apoptosis [53]. Using flow cytometry annexin V staining and Western blotting to detect cleaved caspase-3 and cleaved PARP, we found that RT + BEZ235 significantly increased apoptosis in three CRC cells when compared to RT alone in vitro. The possible reasons why combined BEZ235 and RT treatment induced more apoptosis are as follows. (1) BEZ235 alone can result in apoptosis in certain cancer cell lines like lung cancer, head and neck cancer, sarcoma cells, and colon cancer cells [19,27,54,55]. Our findings showed that BEZ235 alone or BEZ235 + mBEZ235 upregulated the expression of the apoptosis-related molecules cleaved caspase-3 and cleaved PARP. (2) BEZ235 could impair the DNA repair capacities due to RT in the early stage and subsequently cause apoptosis in response to CRC cells. This observation was supported by our in vivo xenograft that showed significant downregulation of p-ATM and p-DNA-PKCs in the RT + BEZ235 group compared to the RT alone group.

Here, we also found that maintenance BEZ235 after RT + BEZ235 significantly increased apoptosis of CRC cells compared to RT + BEZ235 both in vitro and in vivo models based on analyses of flow-cytometry and apoptosis-related markers. Previous studies have revealed that the DNA-DSB repair ATM-signaling pathway was upregulated and persisted for several days after RT, and that upregulated DNA repair signaling may suppress apoptosis of cancer cells [56,57]. Here, BEZ235 maintenance treatment upregulated 53BP1 and γ-H2AX expression, indicating that persistent DNA damage is another mechanism contributing to increased apoptosis and enhancing antitumor effects. Our study demonstrated that maintenance BEZ235 continuously downregulated expression of the mTOR signaling molecules, p-rpS6 and p-eIF4E, suggesting that increased apoptosis may be caused by prolonged attenuation of mTOR signaling. Taken together, our findings suggest that the enhanced radio-sensitizing effect of BEZ235 in CRC cells resulted from increased DNA damage and apoptosis levels via the attenuation of RT-inducing activation of the AKT/mTOR signaling pathway and the impairment of the DNA repair mechanism. Furthermore, the continuous effect of BEZ235 may prolong attenuation of DNA repair capacities, downregulation of mTOR-signaling molecules, persistent DNA damage, and subsequent apoptosis events resulting from the addition of BEZ235 in the RT treatment, and further suppress the tumor growth of CRC cells.

Several studies have attempted to combine second-line chemotherapy or targeted therapy with RT to improve the pCR rate in patients with locally advanced rectal cancer [58,59,60,61]. However, a 6-8-week lag waiting for recovery from acute toxicities may increase the risk of CRC tumor regrowth and metastasis. Our current findings indicate that low-dose targeted maintenance therapy (BEZ235) following RT with BEZ235 treatments prolonged the therapeutic effects on CRC cells both in vitro and in vivo. This could represent another strategy for increasing the pCR rate and improving clinical outcomes in patients with locally advanced rectal cancer.

Although RT with chemotherapy is not the standard treatment for colon cancer, RT may be used in select colon cancer cases, like tumors in sigmoid lesions, tumors that are initially larger than 6 cm in diameter, tumors attached internally to organs after surgery, or tumors not easily removed by surgery alone [62,63]. In addition, two recent studies explored treatment efficacies and the safety of neoadjuvant CCRT in patients with locally advanced colon cancer, showing that oxaliplatin and 5-fluorouracil-based radiotherapy provided increased pCR rates and acceptable adverse effects [64,65]. Our study demonstrates that BEZ235 following local radiation with BEZ235 can efficiently eradicate cancer cells and prolong the treatment effect both in vitro and in vivo. These findings suggest that RT with chemotherapy or BEZ235 might be used as an alternative treatment option for colon cancer patients who have local tumors at sigmoid lesions, locally advanced tumors, or locally adherent tumors after operation.

## 4. Materials and Methods

### 4.1. Cell Lines and Drugs

Considering that p53 mutation and K-RAS mutation status may alter the radiosensitivity [66,67] induced by BEZ235 both in the combination treatment with RT and in the maintenance treatment after RT, we used the following human CRC cell lines: HCT116 (K-RAS mutation; p53 wild type), HT29 (K-RAS wild type; p53 mutation), and SW480 (K-RAS mutation; p53 mutation) to assess the differences in the radiosensitizing effects of BEZ235.

These three CRC cell lines were maintained in Dulbecco’s modified Eagle medium (DMEM) supplemented with 10% fetal bovine serum (Hyclone, Logan, UT, USA), penicillin, and streptomycin (Flow Labs, Rockville, MD, USA). The cells were cultured in an incubator in a humidified atmosphere of 5% CO_2_ at 37 °C. The NVP-BEZ235 (BEZ235) was obtained from Novartis Pharma AG (Basel, Switzerland). BEZ235 was dissolved in dimethyl sulfoxide (Sigma Aldrich, St. Louis, MO, USA) to a stock concentration of 10 mM for the in vitro experiments. For the in vivo experiments, BEZ235 was formulated in NMP/PEG300 (1:9, *v/v*) to the desired concentrations.

A Co^60^ teletherapy unit was used to locally irradiate the desired dose at a dose rate of 1 Gy/min [28]. In the in vitro experiments, three CRC cells were irradiated with 1 Gy, 2 Gy, 3 Gy, or 5 Gy of radiation, and the source-skin-distance was set to 80 cm. In the animal experiments, the xenograft tumor was irradiated with 2 Gy/time. To prevent exposure of the region surrounding the tumor in mice to the radiation beam, a lead block was used.

### 4.2. In Vitro Experiment and Analysis

HCT116 cells (2, 5, or 10 × 10^2^), HT29 cells (2, 5, or 10 × 10^2^), and SW480 (2, 5, or 10 × 10^2^) cells were seeded into 6-well plates and separated into six groups (*n* = 6/plate/group) as follows: (1) control (untreated); (2) BEZ235 alone (10 nM) administered on day 1; (3) radiation alone (5 Gy) given on day 1; (4) combination of radiation (5 Gy) and BEZ235 (10 nM) on day 1, with the radiation given 1 h after drug treatment (RT + BEZ235); (5) maintenance experiments, the same protocol as (4) on day 1, followed by a low dose of BEZ235 (3 nM) administered on day 3 and day 5 (RT + BEZ235 + mBEZ235); and (6) BEZ235 (10 nM) on day 1 followed by maintenance BEZ235 (3 nM) on day 3 and day 5 (BEZ235 + mBEZ235). On days 3 and 5, fresh medium without BEZ235 was added to the control, BEZ235 alone, RT alone, and RT + BEZ235 groups (Figure 1A). After seven days, the number of colonies in each well (clusters of more than 50 cells) was counted using an inverted phase contrast microscope at 100× magnification and photographed.

In order to assess the VEGF-A expression among the groups, 1 × 10^4^ HCT116, HT29, and SW480 cells were seeded into 6-well plates and separated into five groups (*n* = 6/plate/group). The cell culture supernatant was collected every second day, and the concentrations of secretory VEGF-A in the supernatants were determined via enzyme-linked immunosorbent assay (ELISA), according to the manufacturer instructions (R&D System, Minneapolis, MN, USA) [68].

### 4.3. In Vivo Tumor Model

NOD.CB17-Prkdc^scid^ mice (4–5 weeks old) were used in the current study. Animal experimental procedures were approved by the National Taiwan University College of Medicine and College of Public Health Institutional Animal Care and Use Committee (ethical code: No: 20160448 and permission date: January 1, 2017). An aliquot of 1 × 10^6^ HCT116 cells were injected into mouse thighs. Treatment regimens were divided into six groups (six mice/group; Figure 1B) as follows: (1) untreated (control); (2) radiation alone, where 2 Gy radiation was administered on days 1 (D1), 3 (D3), and 5 (D5); (3) BEZ235 alone: 25 mg/kg BEZ235 was administered once daily via oral gavage feeding for five days; (4) BEZ235 (25 mg/kg) was administered once daily via oral gavage for five days, and 2 Gy radiation was administered on D1, D3, and D5, 1 h after BEZ235 treatment (RT + BEZ235); (5) maintenance groups, where the protocol was similar to (4), with the addition of BEZ235 administration (10 mg/kg) once per week for four weeks starting from the second week after the combined treatment (D15, D22, D29, D36) (RT + BEZ235 + mBEZ235); and (6) 25 mg/kg BEZ235 was administered once daily via oral gavage feeding for five days, and the additional administration of BEZ235 (10 mg/kg) on D15, D22, D29, and D36 (BEZ235 + mBEZ235) (Figure 1B). Tumor sizes were measured once per week, and the tumor volume was calculated as π/6 × L × W × H, where L is the length, W is the width, and H is the height. The body weights of the mice were also measured weekly.

### 4.4. Apoptosis Analysis Using Flow Cytometry

The number of apoptotic cells was determined using a FITC Annexin V Dead Cell Apoptosis Kit (V13242, Invitrogen, Waltham, MA, USA). Briefly, after treatment, the cells were washed with PBS and incubated with annexin-binding buffer at a concentration of 1 × 10^6^ cells/mL. Subsequently, the cells were stained with 5 µL of FITC annexin V and 1 µL of propidium iodide in the dark at room temperature for 15 min. The distribution and relative proportion of apoptotic cells were determined using a FACS flow cytometer (Becton-Dickinson, Franklin Lakes, NJ, USA).

### 4.5. Western Blotting Analysis

Proteins from CRC cells and frozen tumor tissues were extracted using Mammalian Protein Extraction Reagent (Pierce, Rockford, IL, USA). Protein extracts were separated in sodium dodecyl sulfate—polyacrylamide gel electrophoresis gels, and then transferred onto polyvinylidene fluoride (PVDF) membranes. Immunoblotting was performed with the following primary antibodies: β-actin (GeneTex; GTX101794; Irvine, CA, USA), caspase-3 (Cell Signaling Technology, #9665; Danvers, MA, USA), cleaved caspase-3 (Asp175; Cell Signaling Technology, #9664), poly (ADP-ribose) polymerase (PARP) (Cell Signaling Technology, #9542), cleaved PARP (Cell Signaling Technology, #5625), eukaryotic translation initiation factor 4E (eIF4E; Cell Signaling Technology, #9742), p-eIF4E (Ser209; Cell Signaling Technology, #9741), S6 ribosomal protein (rpS6; Cell Signaling Technology, #2217), p-rpS6 (Ser235/236; Cell Signaling Technology, #2211), ATM (Cell Signaling Technology, #2873), p-ATM (Ser1981, Cell Signaling Technology, #5883), DNA-PKcs (Cell Signaling Technology, #4602), p-DNA-PKcs (Ser2056; abcam, ab18192; Australia), Ku80 (Cell Signaling Technology, #2753), 53BP1 (Cell Signaling Technology, #4937), Histone H2A.X (Cell Signaling Technology, #7631), p-Histone H2A.X (Ser139; Cell Signaling Technology, #9718), HIF-1α (abcam, ab82832), and VEGF-A (GeneTex, GTX102643) [28,69,70]. The immune complexes were visualized using Western Lightning^®^ Plus-ECL (PerkinElmer Inc., Waltham, MA, USA). The targeted protein was quantified using Image Quant software (GE Healthcare, Buckinghamshire, UK).

### 4.6. Immunofluorescence Analysis of DNA Damage

HCT116, HT29, and SW480 cells were seeded into treated tissue culture glass slides (BD Falcon, 354114) and separated into five treatment groups. After treatment on days 1, 4, and 7, the cells were rinsed three times with PBS and fixed with 3.7% paraformaldehyde at 4 °C for 15 min. Then, the fixed cells were permeabilized with 0.3% Triton X-100 in PBS for 10 min at room temperature. After rinsing with PBS, the cells were blocked with blocking buffer (PBS containing 3% bovine serum albumin and 0.1% Triton X-100) for 1 h at room temperature and incubated separately with the anti-53BP1 (1:100) (Cell Signaling Technology, #4937) and anti-γ-H_2_AX primary antibody (1:200) (Cell Signaling Technology, #9718) at 4 °C overnight. The cells were rinsed three times with PBS, and then incubated with Alexa Fluor 488-conjugated secondary antibodies (1:500) (Invitrogen, Carlsbad, CA, USA) in the dark for 1 h at room temperature. Nuclei were counterstained with DAPI (1:1000 in PBS) (Biotium, Fremont, CA, USA). Images were acquired using an IX71 fluorescence microscope (Olympus) with a 100× oil immersion objective lens and standard FITC filter. Data were collected with CellSens software.

### 4.7. Immunohistochemistry

Tumor specimens from the mice were washed with 1× phosphate buffer solution (PBS), fixed with 4% formaldehyde (Mallinckrodt Chemical Co, St Louis, MO, USA) in PBS, and embedded in paraffin wax. Sections (4 µm) were cut and deparaffinized with antibodies for cleaved caspase-3 (Asp175; Cell Signaling Technology, #9664), p-rpS6 (Ser235/236; Cell Signaling Technology, #2211), p-eIF4E (Ser209; Abcam, Cambridge, UK, ab76256), p-ATM (Ser1981; Abcam, Cambridge, UK, ab81292), p-DNA-PKcs (Ser2056; Abcam, ab18192), 53BP1 (Cell Signaling Technology, #4937), VEGF-A (GeneTex, GTX102643), and HIF-1α (Abcam, ab82832); those antibodies were used as primary antibodies in the indirect immunoperoxidase immunohistochemistry method [28,69,70].

### 4.8. Statistical Analysis

The data from the in vitro experiments (*n* = 6) are presented as the mean ± standard deviation (SD). In animal studies, the results are presented as the mean ± SD for the six mice in each group. Quantification in Western blotting is expressed as the mean ± SD of five experiments. The *p* values of all data were determined using the Student’s *t* test. *p* ˂ 0.05 was considered statistically significant.

## 5. Conclusions

**Figure 7:**
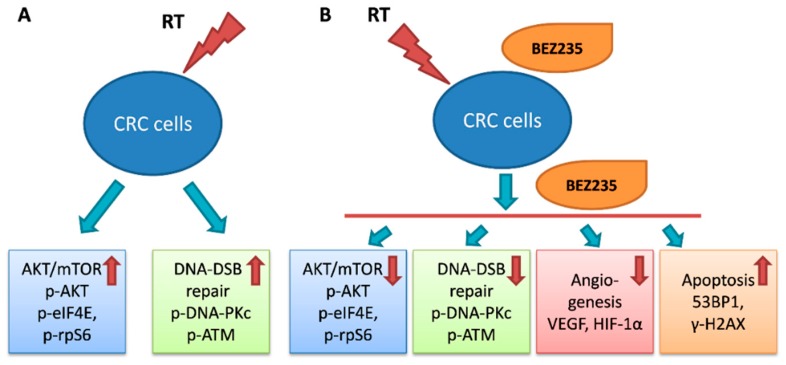
Maintenance BEZ235 treatment after BEZ235 combined with radiation treatment reduced cell viability and inhibited tumor growth in a colorectal cancer cell model. (**A**) Although radiotherapy (RT) inhibited tumor growth of colorectal cancer (CRC) cells, RT also upregulated the expression of AKT/mTOR signaling molecules (p-AKT, p-eIF4E, and p-rpS6) and DNA double-strand break (DSB) repair-related molecules (p-ATM and p-DNA-PKcs). (**B**) Maintenance BEZ235 treatment prolonged the therapeutic effect of concurrent BEZ235 + RT treatment by attenuating mTOR signaling activation, impairing the DNA-DSB repair mechanism, inhibiting angiogenesis (VEGF-A and HIF-1α), and enhancing apoptosis via cleaved caspase-3, cleaved PARP, and radiation-induced DNA damage (γ-H2AX and 53BP1).

Our results indicate that maintenance BEZ235 treatment following BEZ235 + RT treatment reduced cell viability and inhibited tumor growth in a CRC cell xenograft tumor model. The key mechanisms of maintenance BEZ235 treatment in prolonging the therapeutic effect of concurrent BEZ235 + RT treatment occurred through the attenuation of mTOR signaling activation, impairment of the DNA-DSB repair mechanism, and inhibition of angiogenesis (Figure 7). Since a more satisfactory pCR rate and a higher anal preservation rate can be achieved in rectal cancer by using neoadjuvant CCRT followed by a delayed operation, our findings revealed that maintenance BEZ235 treatment may prolong the therapeutic effect of neoadjuvant RT with or without chemotherapy or BEZ235. Furthermore, it can prevent micrometastases during the period of 6–8 weeks without treatment before operating. Additional clinical studies evaluating the efficacy of this treatment strategy in patients with locally advanced rectal cancer are warranted [71,72].

## Supplementary Materials

The following are available online at https://www.mdpi.com/2072-6694/11/8/1204/s1, Figure S1: The colony formation study with 200 and 500 cells seeded per well; Figure S2: Flow cytometry apoptosis analysis by annexin V staining; Figure S3: Expressions of apoptosis-, mTOR signaling pathway-, angiogenesis-, DNA-DSB repair-, and DNA damage-related molecules in BEZ235+mBEZ235 treatment group; Figure S4: Maintenance BEZ235 treatment significantly inhibited CRC xenograft tumor growth when compared to control group; Figure S5: Expression of apoptosis-, mTOR signaling pathway-, DNA-DSB repair-, DNA damage-, and angiogenesis-related molecules in CRC xenograft tissue following BEZ235+mBEZ235 treatment by immunohistochemistry; Figure S6: Protein expression levels following each treatment were evaluated in HCT116 xenograft tissue via western blotting.
