# Supplementary material for: Maintenance BEZ235 Treatment Prolongs the Therapeutic Effect of the Combination of BEZ235 and Radiotherapy for Colorectal Cancer
Source: Cancers (Basel). 2019 Aug 19;11(8):1204. doi: 10.3390/cancers11081204 (PMC6721476; doi:10.3390/cancers11081204)
Supplement: Supplementary file 1 [file cancers-11-01204-s001.zip › Supplementary Figure 1 to 6/Supplementary Figure 1 cancers-485053.pdf]

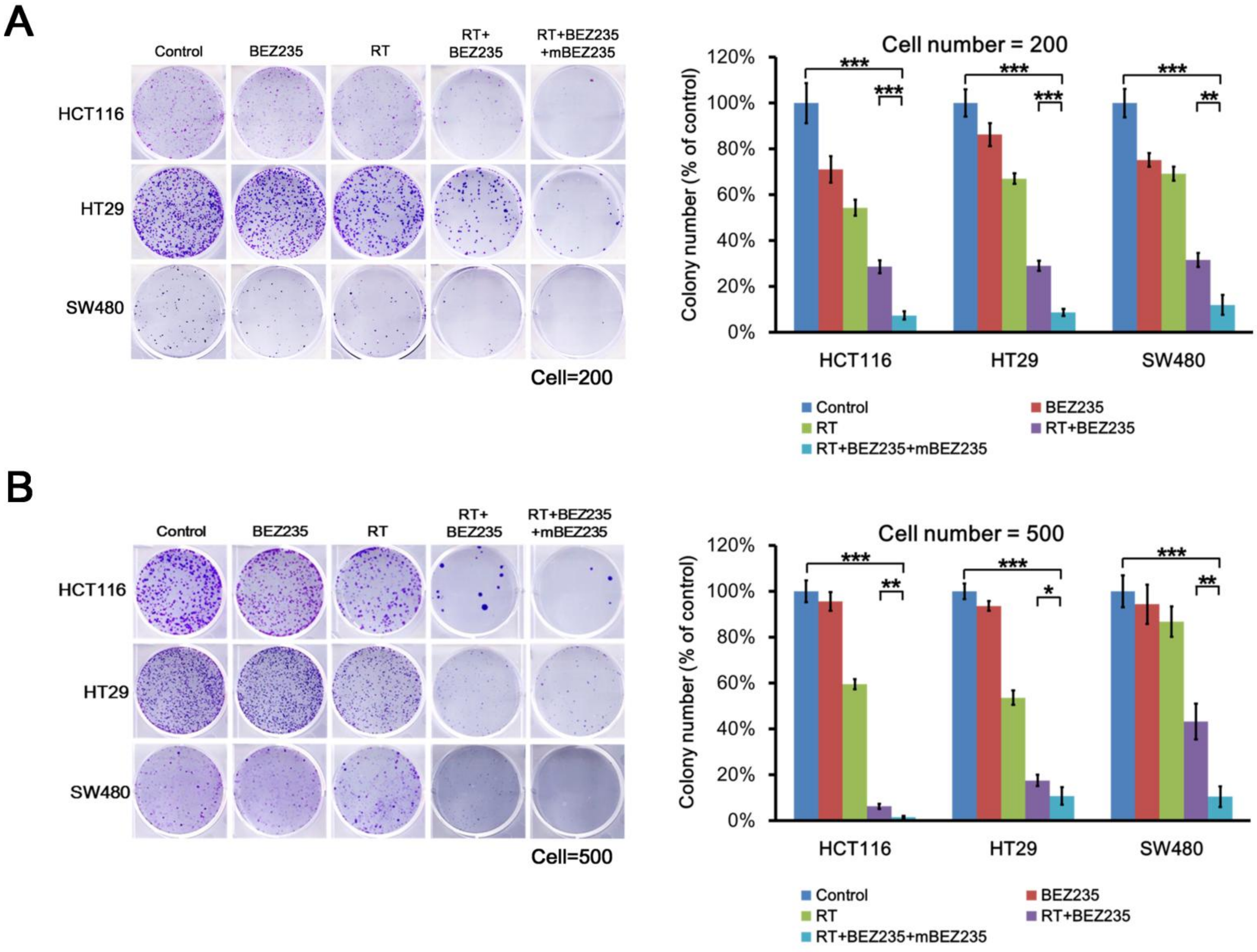

Supplementary Figure 1. (A) The colony formation study with 200 and 500 cells seeded per well, showed a similar trend that RT+BEZ235+mBEZ235 treatment significantly inhibited cell survival compared with the other four treatments. (B) Enumeration of colonies of three CRC cell lines with various seeded cell numbers. The relative colony ratio of the control group was defined as 100%. The results are expressed as the mean ± standard error (SE) of three experiments. **P* < 0.05; ***P* < 0.01; ****P* < 0.001.
