# Supplementary material for: Maintenance BEZ235 Treatment Prolongs the Therapeutic Effect of the Combination of BEZ235 and Radiotherapy for Colorectal Cancer
Source: Cancers (Basel). 2019 Aug 19;11(8):1204. doi: 10.3390/cancers11081204 (PMC6721476; doi:10.3390/cancers11081204)
Supplement: Supplementary file 1 [file cancers-11-01204-s001.zip › Supplementary Figure 1 to 6/Supplementary Figure 2 cancers-485053.pdf]

A

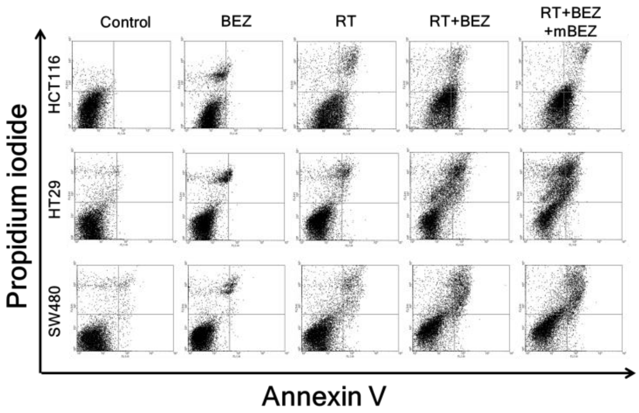

	Annexin V	Control	BEZ	RT	RT+BEZ	RT+BEZ +mBEZ
HCT116	Alive cells	90.69±1.41	81.61±2.3	68.61±1.55	59.65±4.98	46.98±5.49
	Early apoptosis	0.79±0.51	1.55±0.68	12.71±1.33	14.7±1.58	29.8±1.72
	Late apoptosis	0.44±0.32	1.21±0.48	8.6±1.87	10.41±1.08	14.54±2.37
	Necrosis	8.08±1.54	15.62±1.97	10.08±1.67	15.24±2.81	8.68±2.07
HT29	Alive cells	89.6±2.36	82.54±1.92	79.56±2.72	58.46±3.53	38.16±3.63
	Early apoptosis	0.58±0.16	0.8±0.62	1.71±0.84	3.63±1.27	3.81±1.31
	Late apoptosis	1.38±0.82	4.67±1.55	6.18±1.96	15.3±2.39	20.82±1.79
	Necrosis	8.44±2.83	11.99±2.49	12.55±2.53	22.61±2.69	37.21±3.04
SW480	Alive cells	92.99±3.24	81.35±4.08	75.56±4.1	61.89±3.39	55.73±5.22
	Early apoptosis	0.83±0.54	0.54±0.32	3.26±1.29	4.09±1.61	6.77±0.79
	Late apoptosis	1.62±0.46	5.17±1.55	8.31±0.45	17.28±0.89	21.26±1.67
	Necrosis	4.55±2.32	12.93±2.38	12.86±3.27	16.74±1.84	16.25±2.84

B

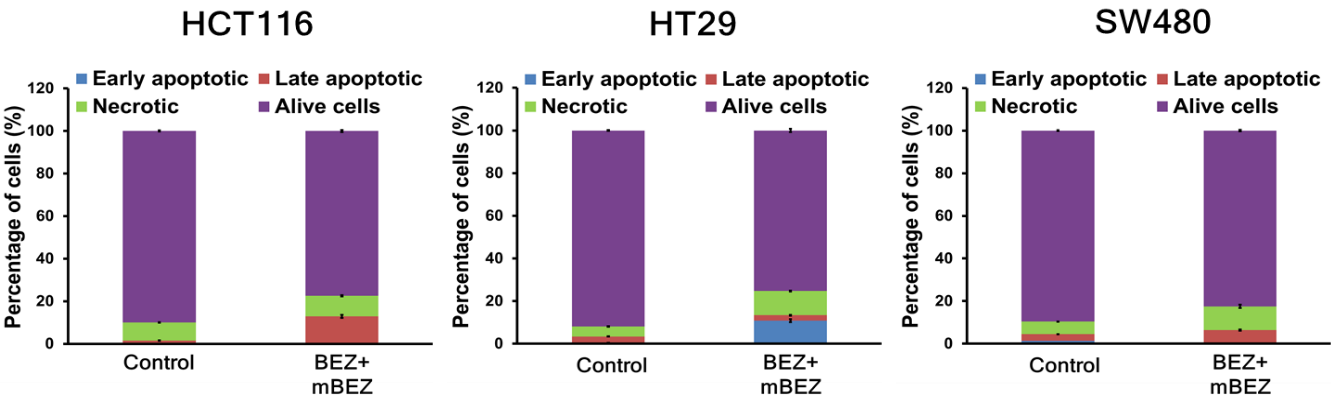

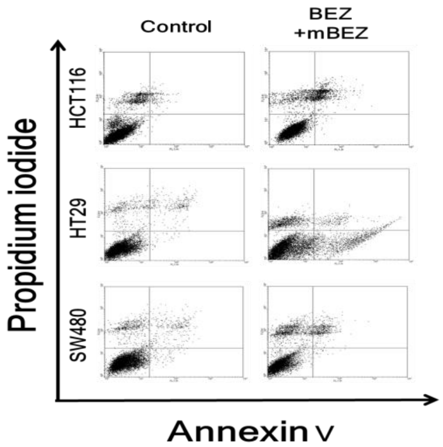

	Annexin V	Control	BEZ+ mBEZ
HCT116	Alive cells	89.93±0.23	77.51±0.49
	Early apoptosis	0.06±0.02	0.26±0.05
	Late apoptosis	1.5±0.13	12.63±0.72
	Necrosis	8.51±0.13	9.6±0.29
HT29	Alive cells	91.94±0.15	75.3±0.78
	Early apoptosis	0.37±0.07	10.83±0.66
	Late apoptosis	3.03±0.04	2.52±0.25
	Necrosis	4.66±0.13	11.35±0.28
SW480	Alive cells	89.6±0.22	82.46±0.38
	Early apoptosis	1.31±0.3	0.36±0.09
	Late apoptosis	3.16±0.07	5.99±0.31
	Necrosis	5.93±0.09	11.19±0.74

Supplementary Figure 2. (A) Flow cytometry apoptosis analysis by annexin V staining of HCT116, HT29, and SW480 cells following control, BEZ235 (BEZ), RT, RT+BEZ, and RT+BEZ+mBEZ treatments. Summary of the annexin V-FITC apoptosis assay results showing the percentages of early apoptotic, late apoptotic, necrotic, and living cells. The results are expressed as the mean ± SE of three experiments from each of the three CRC cell lines. Annexin V staining revealed that RT+BEZ+mBEZ treatment resulted in more predominant apoptosis than BEZ, RT, RT+BEZ, and control treatments. (B) Flow cytometry apoptosis analysis by annexin V staining of HCT116, HT29, and SW480 cells following control and BEZ+mBEZ treatments. Annexin V staining revealed that BEZ+mBEZ treatment resulted in more predominant apoptosis than control treatments.
