# Supplementary material for: Maintenance BEZ235 Treatment Prolongs the Therapeutic Effect of the Combination of BEZ235 and Radiotherapy for Colorectal Cancer
Source: Cancers (Basel). 2019 Aug 19;11(8):1204. doi: 10.3390/cancers11081204 (PMC6721476; doi:10.3390/cancers11081204)
Supplement: Supplementary file 1 [file cancers-11-01204-s001.zip › Supplementary Figure 1 to 6/Supplementary Figure 3 cancers-485053.pdf]

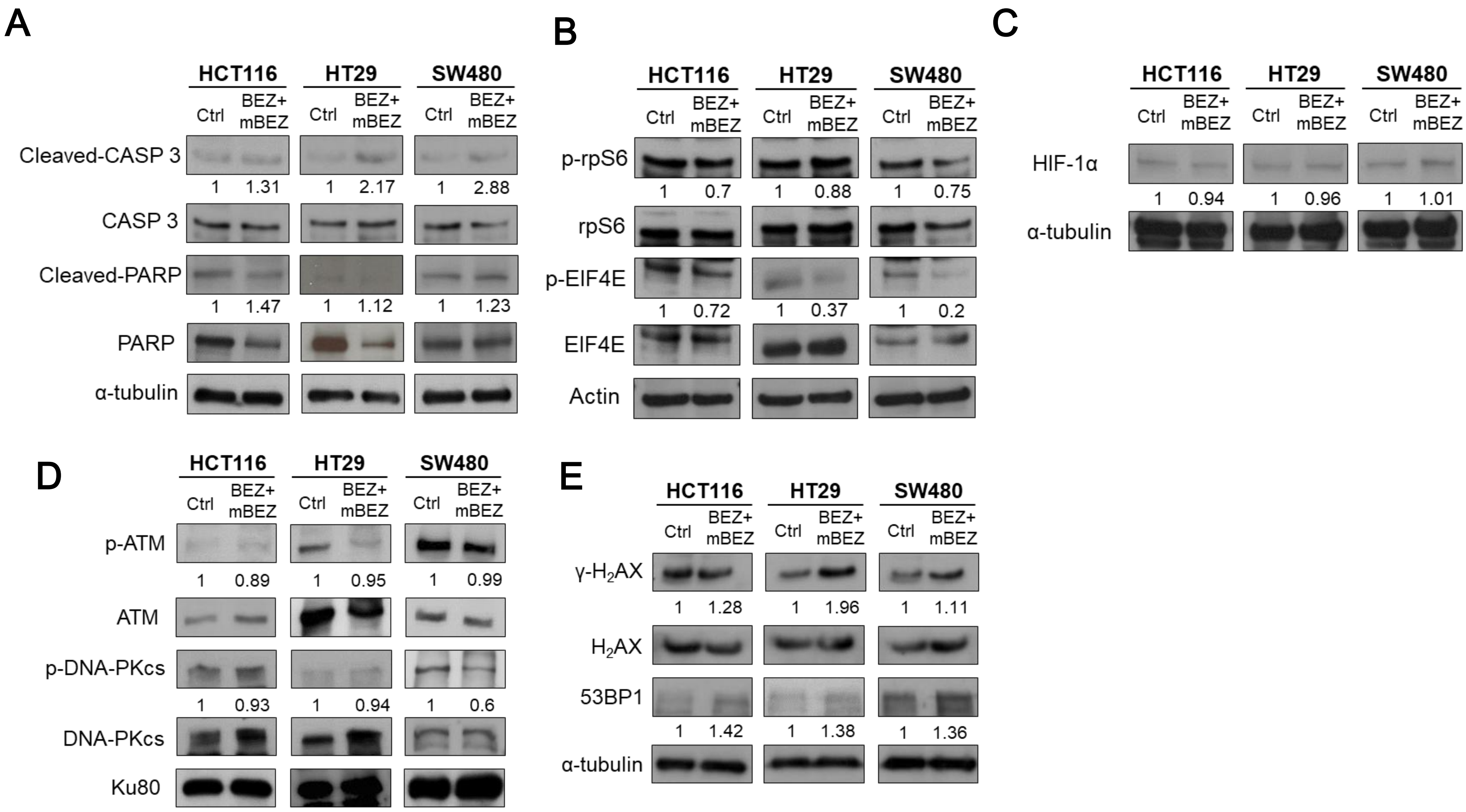

Supplementary Figure 3. BEZ235+mBEZ235 treatment (A) increased expression of apoptosis-related molecules, cleaved caspase 3 [CASP 3] and cleaved PARP (B) attenuated mTOR signaling molecules expression (p-rpS6 and p-eIF4E) (C) did not alter expression of angiogenesis-associated molecules, HIF-1α (D) slightly downregulated expression of p-ATM and DNA-PKcs (DNA-repair) (E) increased DNA damage-related molecules, 53BP1 and γ-H2AX.
