# Supplementary material for: Maintenance BEZ235 Treatment Prolongs the Therapeutic Effect of the Combination of BEZ235 and Radiotherapy for Colorectal Cancer
Source: Cancers (Basel). 2019 Aug 19;11(8):1204. doi: 10.3390/cancers11081204 (PMC6721476; doi:10.3390/cancers11081204)
Supplement: Supplementary file 1 [file cancers-11-01204-s001.zip › Supplementary Figure 1 to 6/Supplementary Figure 4 cancers-485053.pdf]

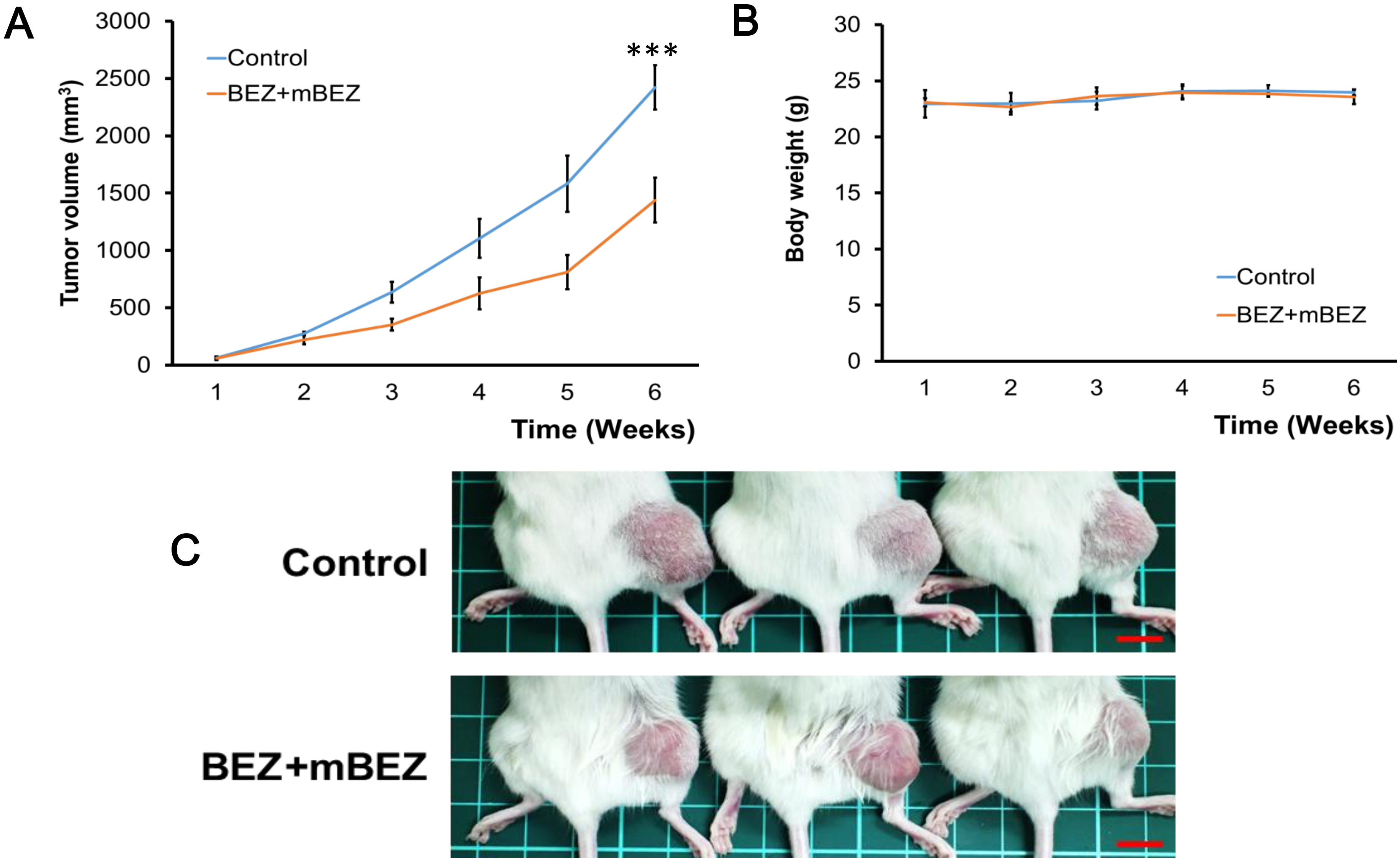

Supplementary Figure 4. Maintenance BEZ235 treatment significantly inhibited CRC xenograft tumor growth when compared to control group. (A) Maintenance BEZ235 following BEZ235 (BEZ+mBEZ) treatment significantly suppressed xenograft tumor growth compared with control groups. The results of the tumor volume are expressed for n = 6 in each treatment group. *** $P < 0.001$. (B) The body weight of mice in the two groups did not differ, and no marked changes were observed during the treatment period. (C) Representative pictures of each treatment group. Mice were killed during the 6th week after treatment. Scale bar: 10 mm.
