# Supplementary material for: Maintenance BEZ235 Treatment Prolongs the Therapeutic Effect of the Combination of BEZ235 and Radiotherapy for Colorectal Cancer
Source: Cancers (Basel). 2019 Aug 19;11(8):1204. doi: 10.3390/cancers11081204 (PMC6721476; doi:10.3390/cancers11081204)
Supplement: Supplementary file 1 [file cancers-11-01204-s001.zip › Supplementary Figure 1 to 6/Supplementary Figure 5 cancers-485053.pdf]

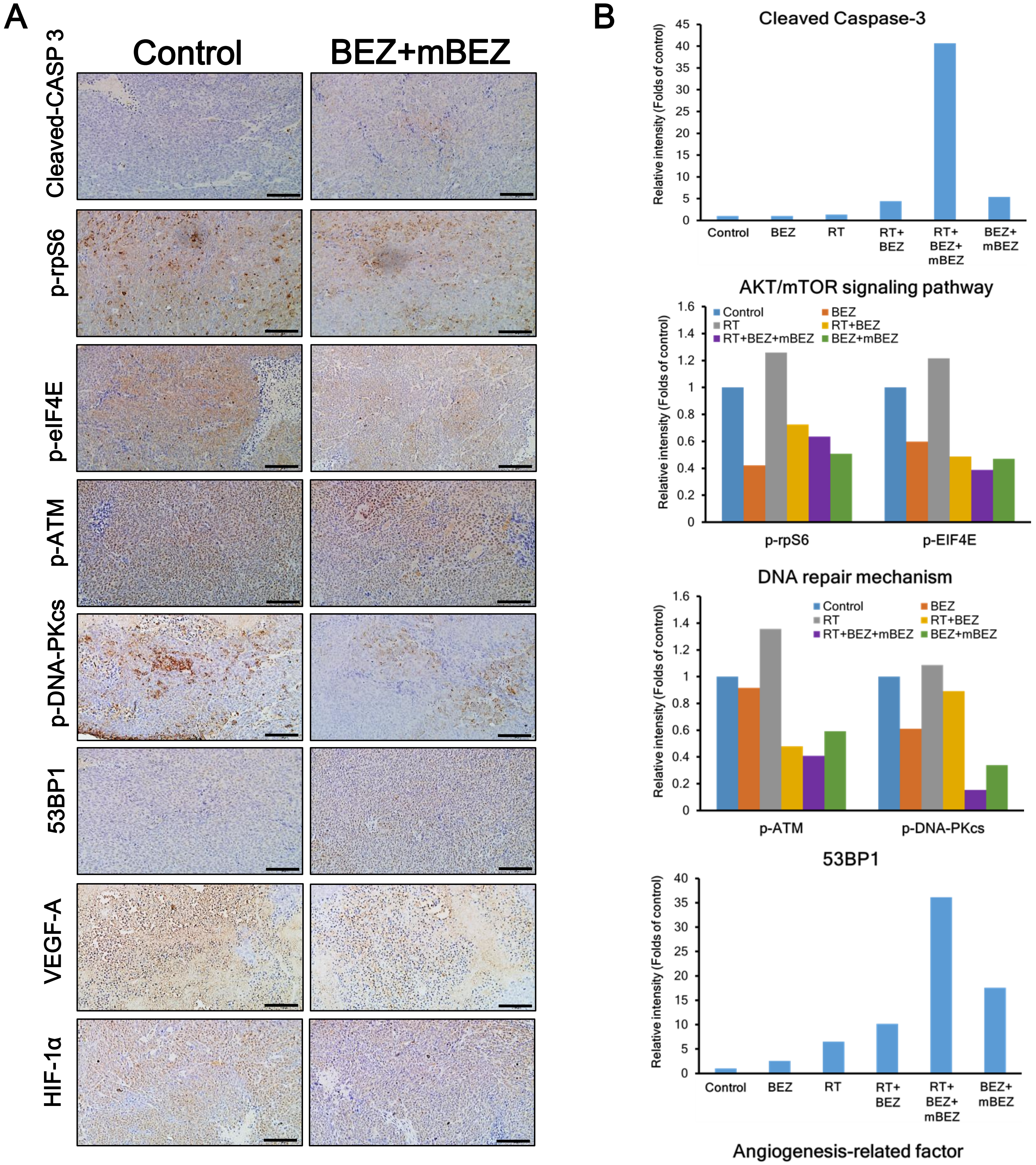

Supplementary Figure 5. (A) Expression of apoptosis-, mTOR signaling pathway-, DNA-DSB repair-, DNA damage-, and angiogenesis-related molecules in CRC xenograft tissue following BEZ235+mBEZ235 treatment and control by immunohistochemistry. (B) The quantification of expression level of different molecules in tumor cells of each group of HCT116 xenograft tumor by immunohistochemical staining: the expression level of cleaved caspase 3, p-rpS6, p-eIF4E, p-ATM, p-DNA-PKcs, 53BP1, VEGF-A and HIF-1α in each group.
