# Supplementary material for: Maintenance BEZ235 Treatment Prolongs the Therapeutic Effect of the Combination of BEZ235 and Radiotherapy for Colorectal Cancer
Source: Cancers (Basel). 2019 Aug 19;11(8):1204. doi: 10.3390/cancers11081204 (PMC6721476; doi:10.3390/cancers11081204)
Supplement: Supplementary file 1 [file cancers-11-01204-s001.zip › Supplementary Figure 1 to 6/Supplementary Figure 6 cancers-485053.pdf]

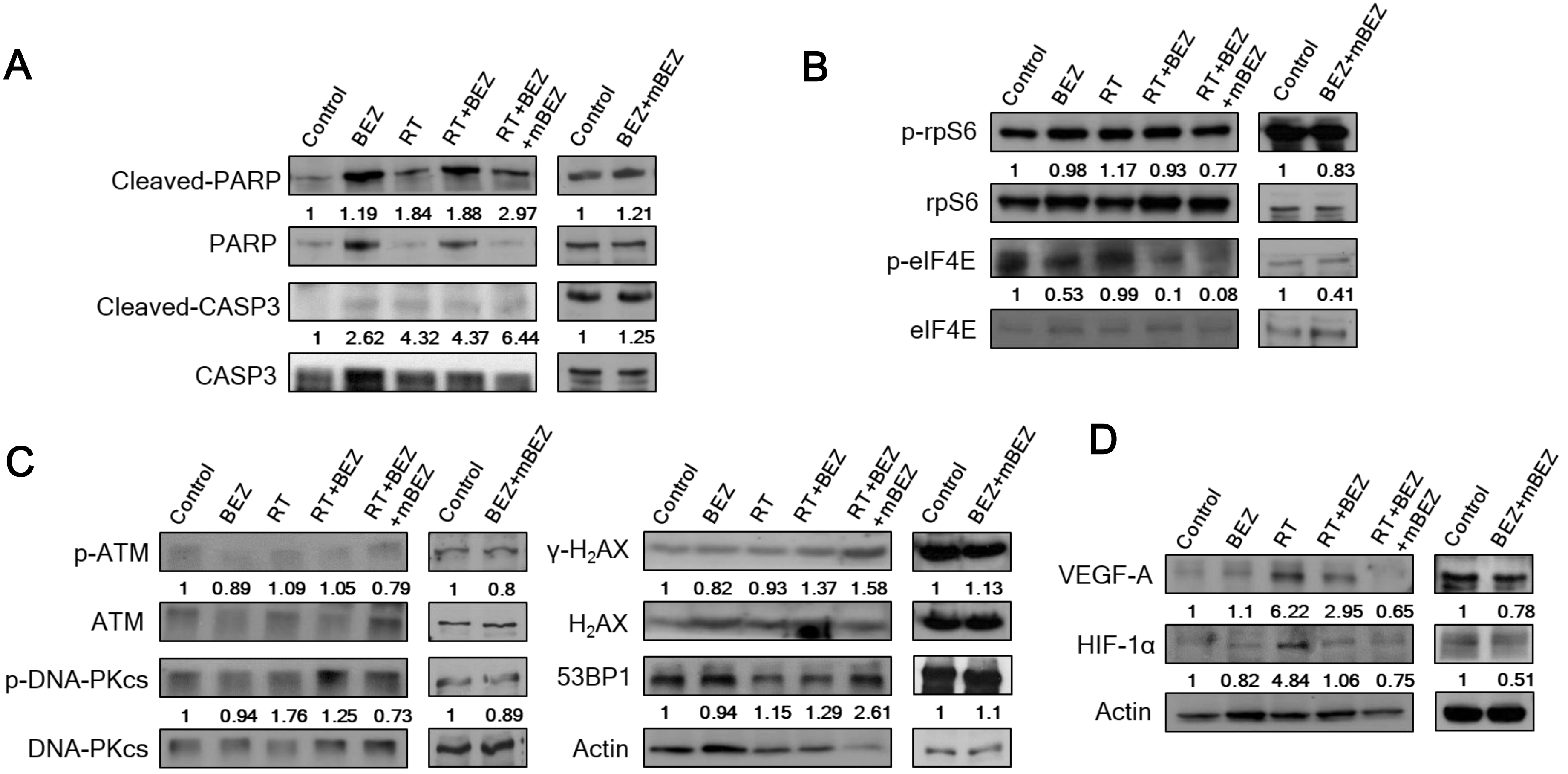

Supplementary Figure 6. Protein expression levels following each treatment were evaluated in HCT116 xenograft tissue via western blotting. The expression levels of (A) cleaved PARP and cleaved caspase 3 (CASP3); (B) p-rps6 and p-eIF4E; (C) p-ATM, p-DNA-PKcs, γ-H2AX, and 53BP1; and (D) VEGF-A and HIF-1α following each treatment, including control, BEZ235 (BEZ), RT, RT+BEZ, RT+BEZ+mBEZ, and BEZ+mBEZ groups.
